# Pre-contrast T1-weighted imaging of the spinal cord may be unnecessary in patients with multiple sclerosis

**DOI:** 10.1007/s00330-021-08077-4

**Published:** 2021-06-09

**Authors:** Isabelle Riederer, Mark Mühlau, Claus Zimmer, Magaly Gutbrod-Fernandez, Nico Sollmann, Jan S Kirschke

**Affiliations:** 1 Department of Diagnostic and Interventional Neuroradiology, School of Medicine, Klinikum rechts der Isar, Technical University of Munich, Ismaninger Str. 22, 81675 Munich, Germany; 2 TUM-Neuroimaging Center, Klinikum rechts der Isar, Technical University of Munich, Munich, Germany; 3 Department of Neurology, School of Medicine, Klinikum rechts der Isar, Technical University of Munich, Ismaninger Str. 22, 81675 Munich, Germany; 4 Department of Diagnostic and Interventional Radiology, University Hospital Ulm, Albert-Einstein-Allee 23, 89081 Ulm, Germany

**Keywords:** Multiple sclerosis, Magnetic resonance imaging, Spinal cord, Contrast media

## Abstract

**Objectives:**

Multiple sclerosis (MS) is an inflammatory disease frequently involving the spinal cord, which can be assessed by magnetic resonance imaging (MRI). Here, we hypothesize that pre-contrast T1-w imaging does not add diagnostic value to routine spinal MRI for the follow-up of patients with MS.

**Methods:**

3-T MRI scans including pre- and post-contrast T1-w as well as T2-w images of 265 consecutive patients (mean age: 40 ± 13 years, 169 women) with (suspected) MS were analyzed retrospectively. Images were assessed in two separate reading sessions, first excluding and second including pre-contrast T1-w images. Two independent neuroradiologists rated the number of contrast-enhancing (ce) lesions as well as diagnostic confidence (1 = unlikely to 5 = very high), overall image quality, and artifacts. Results were compared using Wilcoxon matched-pairs signed-rank tests and weighted Cohen’s kappa (*κ*).

**Results:**

Fifty-six ce lesions were found in 43 patients. There were no significant differences in diagnostic confidence between both readings for both readers (reader 1: *p* = 0.058; reader 2: *p* = 0.317). Inter-rater concordance was both moderate regarding artifacts (*κ* = 0.418) and overall image quality (*κ* = 0.504). Thirty-one black holes were found in 25 patients with high diagnostic confidence (reader 1: 4.04 ± 0.81; reader 2: 3.80 ± 0.92) and substantial inter-rater concordance (*κ* = 0.700).

**Conclusions:**

Availability of pre-contrast T1-w images did not significantly increase diagnostic confidence or detection rate of ce lesions in the spinal cord in patients with MS. Thus, pre-contrast T1-w sequences might be omitted in routine spinal MRI for follow-up exams, however not in special unclear clinical situations in which certainty on contrast enhancement is required.

**Key Points:**

- *Availability of pre-contrast T1-w images does not increase diagnostic confidence or detection rate of contrast-enhancing lesions in the spinal cord of MS patients.*
- *Excluding pre-contrast T1-w sequences reduces scan time, thus providing more time for other sequences or increasing the patients’ compliance.*

## Introduction

Multiple sclerosis (MS) is a chronic autoimmune inflammatory disease of the central nervous system (CNS) that frequently involves the spinal cord. Signal alterations in the spinal cord according to magnetic resonance imaging (MRI) can be found in up to 80–92% [1–3] of the patients with MS and are frequently located in the cervical spinal cord (56.4%) [2]. Accordingly, diagnostic criteria for MS corresponding to the 2017 revised McDonald Criteria [4] and the MAGNIMS consensus guidelines [5] include the spinal cord as a specific anatomical location for the evaluation of dissemination in space (DIS).

However, MRI of the spinal cord is still challenging due to the long and thin structure of the spinal cord, requiring to cover a large volume with high spatial resolution. Despite recent developments in accelerating image acquisitions, MRI of the spinal cord is still time-consuming and can, therefore, not be tolerated by all patients and can occupy a considerable amount of available scan time. Consequences related to long scan times include motion artifacts, early abort of the examination, and may result in non-compliance for future examinations.

Standard MRI of the spinal cord in case of (suspected) MS includes T2-w sequences in sagittal and axial orientations. Additional contrast-enhanced T1-w sequences can be obtained to evaluate a new clinical attack or to assess dissemination in time (DIT), whereas its role for the fulfillment of DIT is limited [5] and only a small percentage of spinal cord lesions enhance [6]. Current guidelines for MRI of the spinal cord recommend at least two sequences: T2-w and short tau inversion recovery (STIR), T2-w and double inversion recovery (DIR), or T2-w and post-contrast T1-w sequences or the performance of spinal cord imaging directly after contrast-enhanced brain MRI to reduce additional contrast administration [5–7].

As standard, a T1-w sequence is performed before and after the administration of contrast medium for proper assessment of a possible contrast enhancement (CE) and for excluding other pathologies leading to an increased signal. Bot and Barkhof already suggested that additionally to T2-w images, sagittal post-contrast T1-w images of the spinal cord may be sufficient [8]. However, they did not provide scientific evidence for this suggestion, but pointed out that MS-typical pathologies of the spinal cord are rarely seen in pre-contrast T1-w images, contrary to the black holes in T1-w images of the brain in MS patients.

Therefore, the purpose of this study was to investigate whether pre-contrast T1-w imaging is needed in imaging of the spinal cord of patients with MS. We hypothesize that sparing dedicated pre-contrast T1-w imaging would not lead to significant decreases in diagnostic confidence for the detection of spinal lesions.

## Methods

### Patients

The study cohort consisted of 265 consecutive patients (169 women and 96 men; mean age: 40 ± 13 years; age range: 18–79 years) with known MS or symptoms suggestive for MS, who had undergone spinal MRI in the clinical routine setting between January 2018 and August 2019. All MRI data were acquired at one center and the patients were included in a prospective MS cohort. Final diagnoses were established by the treating neurologists considering the combination of clinical history, symptoms, imaging findings, and paraclinical tests.

Data were analyzed retrospectively with the approval of the local ethics committee. Requirement for written informed consent was waived by the institutional review board due to the retrospective character of this study.

### MRI acquisition

Imaging was performed on 3-T scanners (Ingenia (*n* = 140 patients), Achieva dStream (*n* = 98 patients), Ingenia Elition X (*n* = 3 patients), Philips Healthcare; and Magnetom Verio (*n* = 24 patients), Siemens Healthineers) using a body coil. All MRI included 2D T2-w turbo spin echo (TSE) sequences in sagittal and axial orientation and sagittal 2D T1-w TSE sequences before and after the administration of gadolinium (Gd) with total scan times ranging between 16.9 and 29.2 min (Ingenia: 16.9 min, Achieva dStream: 20.0 min, Ingenia Elition X: 22.2 min, and Verio: 29.2 min; Table 1). In cases of suspected MS lesions, dedicated segment-wise axial T1-w imaging after contrast administration was added. Sagittal scans had a slice thickness of 2 mm and axial scans of 4 mm. The delay between the administration of contrast agent and post-contrast T1-w imaging in sagittal orientation was in median 6.2 min (range: 4.0–8.5 min).

**Table 1:** MRI acquisition parameters for the T1-w and T2-w sequences in sagittal orientation for each scanner

	Ingenia		Achieva dStream		Ingenia Elition X		Verio	
	T1 TSE	T2 TSE	T1 TSE	T2 TSE	T1 TSE	T2 TSE	T1 TSE	T1 TSE
Acquisition matrix	299 × 300	316 × 285	312 × 250	328 × 240	377 × 268	355 × 258	512 × 512	384 × 288
TR (ms)	450	2800	599	3584	574	3000	680	3000
TE (ms)	17	120	8	100	8	110	12	107
Acquisition time (s)	139	112	229	208	295	210	240	190
Sections	15	15	15	15	15	15	15	15
Slice thickness (mm)	2	2	2	2	2	2	2	2

### MRI analysis

Data were assessed twice and independently by two neuroradiologists (reader 1: 8.5 years and reader 2: 3 years of experience in image reading of MS lesions), blinded to diagnosis and symptoms, on a standard picture archiving and communication system (PACS) workstation (Sectra Workstation IDS7, Sectra AB). The interval between both readings was 4 weeks to minimize recall bias, and the readers were blinded to the readings of each other.

In the first reading session, post-contrast T1-w images were analyzed together with sagittal and axial T2-w images. Both readers counted the number of contrast-enhancing (ce) lesions in the spinal cord. Furthermore, qualitative features were rated on 3- to 5-point Likert scales regarding diagnostic confidence for lesion detection (1: unlikely, 2: vague, 3: likely, 4: high, or 5: very high), overall image quality (1: very good to perfect, 2: good to very good, 3: medium, 4: poor, or 5: inappropriate), visual CE (1: minimal, 2: moderate, or 3: strong), and artifacts in T1-w images (1: none, 2: unclear, 3: mild, or 4: strong). In the second reading, sagittal pre- and post-contrast T1-w images were analyzed together with sagittal and axial T2-w images regarding the number of ce lesions and diagnostic confidence on a Likert scale identical to the first reading.

Afterwards, a consensus read was done in patients with discrepant numbers of ce lesions. A ce lesion was defined as a circumscribed hyperintense signal within the spinal cord in post-contrast T1-w imaging, with a corresponding and spatially overlapping hyperintense signal on T2-w imaging.

Furthermore, both neuroradiologists counted black holes in pre-contrast T1-w images and gave their diagnostic confidence on a Likert scale as mentioned above. Afterwards, a consensus read was performed. A black hole was defined as a well-defined T1-hypointense lesion in pre-contrast T1-w imaging with surrounding normal tissue in the spinal cord and with corresponding hyperintense signal on T2-w imaging.

Additionally, both readers counted and categorized all hyperintense lesions on T2-w imaging in consensus as follows: localization at a cervical versus thoracic vertebral level, localization predominantly in gray matter versus white matter, circumscribed versus longitudinal extensive appearance (longer than two vertebral bodies).

### Statistical analyses

Statistical analyses were performed using SPSS 25 (SPSS Statistics for Windows, IBM Corp.). Descriptive statistics including mean and standard deviation were calculated for cohort demographics and the scores derived from image reading. The number of lesions and fraction of patients presenting with lesions are given as absolute and/or relative frequencies.

Wilcoxon matched-pairs signed-rank tests were conducted to compare overall image quality, visual CE, and artifacts between both readers. Additionally, Wilcoxon matched-pairs signed-rank tests were performed to compare diagnostic confidence, number of ce lesions, and sensitivity and specificity between both reading sessions for each reader separately. Intra- and inter-rater concordance were assessed by using weighted Cohen’s kappa (*κ*). A value of *p* < 0.05 was considered statistically significant.

## Results

Final diagnoses for the 265 patients established by the treating neurologists included suspected or definite MS (*n* = 203), clinically isolated syndrome (CIS; *n* = 41), radiologically isolated syndrome (RIS; *n* = 3), neuromyelitis optica spectrum disorders (NMOSD; *n* = 8), psychosomatic disorders (*n* = 5), or remained unclear (*n* = 5). MRI of the patients was performed for routine follow-up (*n* = 71), because of new symptoms (*n* = 94), or for the initial diagnosis of MS (*n* = 100).

Overall, 183/265 (69%) patients showed 745 T2-hyperintense lesions in the spinal cord (Table 2), with 395/745 (53%) lesions being located in the cervical and 350/745 (47%) lesions being located in the thoracic spinal cord. 255/745 (34%) lesions were predominantly located centrally in the gray matter and 490/745 (66%) lesions were predominantly located peripherally in the white matter. 696/745 (93%) lesions had a circumscribed appearance and 49/745 (7%) showed a longitudinally extensive configuration of at least two vertebral heights. None of the patients with psychosomatic disorders or unclear diagnoses (*n* = 10) showed a pathological hyperintense signal in the spinal cord on T2-w images.

**Table 2:** Number of patients and lesions listed separately per diagnosis

Diagnosis	Number of patients	Number of patients with lesions	Number of T2 lesions	Number of patients with contrast-enhancing lesions	Number of contrast-enhancing lesions
MS	203	151	629	37	49
CIS	41	21	65	5	6
RIS	3	3	27	0	0
NMOSD	8	8	24	1	1
Others	10	0	0	0	0
Sum	265	183	745	43	56

**Fig. 1:**
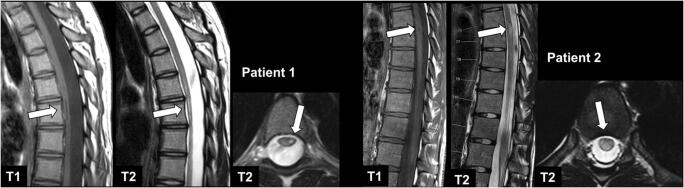
MRI of a 19-year-old female patient with NMOSD (patient 1) and a 20-year-old female patient with relapsing-remitting MS (patient 2) with sagittal T1-w, sagittal T2-w, and axial T2-w images (from left to right). Note the long-segment T1-w hypointense lesion in the thoracic spinal cord on the left side of patient 1 with concomitant atrophy of the spinal cord and the focal T1-w hypointense lesion in patient 2 (arrows), compatible with black holes

**Fig. 2:**
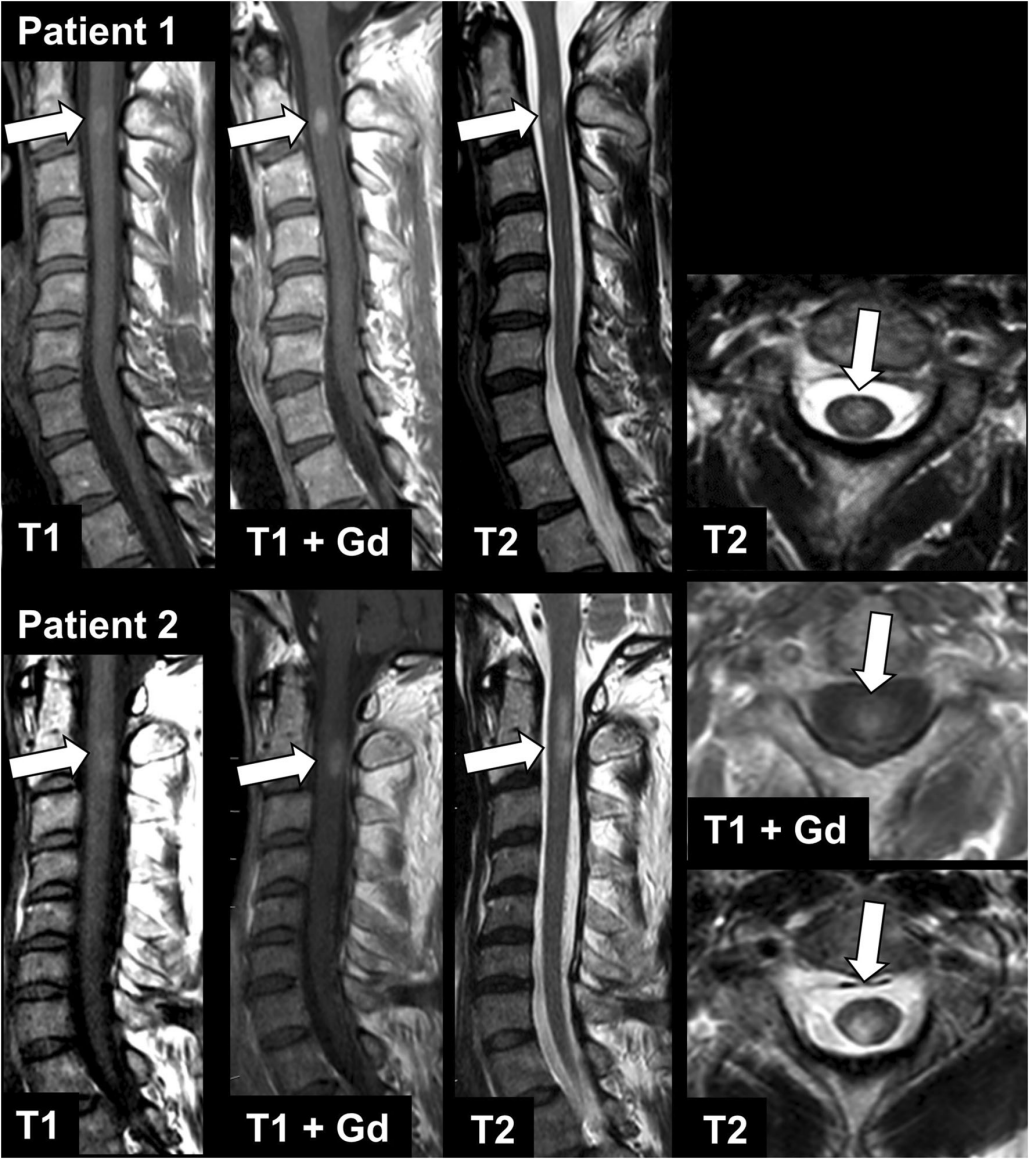
MRI of two patients with MS (patient 1: 39-year-old female, patient 2: 48-year-old male) with sagittal pre- and post-contrast T1-w, sagittal T2-w, axial post-contrast T1-w (not available for patient 1), and axial T2-w images. Note the lesions in the cervical spinal cord at level C2 with slight hyperintense signal in pre-contrast T1-w images and strong contrast enhancement (arrows)

In consensus reading, 31 black holes (Fig. 1) were found in 25 patients (reader 1: 21 lesions in 17 patients; reader 2: 28 lesions in 24 patients). Diagnostic confidence was rated as high by both readers (reader 1: 4.04 ± 0.81; reader 2: 3.80 ± 0.92). Inter-rater concordance was substantial (*κ* = 0.700). A typical hypointense artifact on T1-w imaging was found by both readers in the cervical spinal cord at level C5/C6, most probably caused by swallowing, without a corresponding hyperintense signal on T2-w images. Only two lesions in the cervical spinal cord appeared hyperintense on pre-contrast T1-w images, together with strong CE on respective post-contrast T1-w images (Fig. 2).

**Fig. 3:**
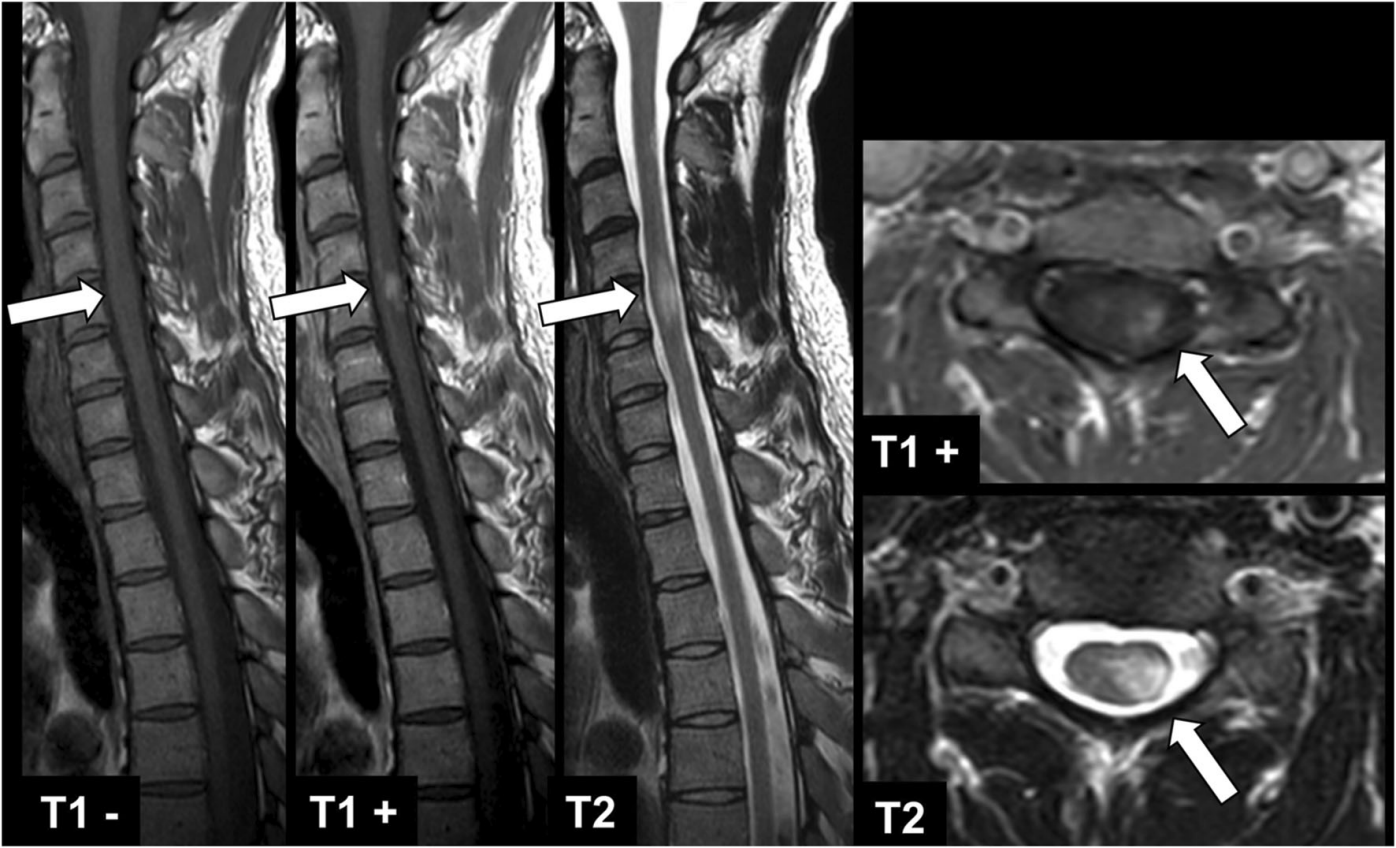
MRI of a 34-year-old male patient with MS with sagittal pre- and post-contrast T1-w, sagittal T2-w, axial post-contrast T1-w, and axial T2-w images. Note the typical contrast-enhancing lesion in the cervical spinal cord at level C5 with isointense signal in pre-contrast T1-w images, hyperintense signal in T2-w images, and strong contrast enhancement (arrows)

In consensus reading, 56 ce lesions (Fig. 3) were counted in 43 patients (first/second reading: reader 1: 56/56; reader 2: 55/56). More details about numbers of ce lesions are presented in Table 3, separately for each session and reader. Reader 1 showed a sensitivity of 98.2% or 97.7% and a specificity of 99.9% or 99.3% for both sessions when counting the number of lesions or number of patients with lesions, respectively. Reader 2 showed a sensitivity of 92.9% or 94.6% and a specificity of 99.6% when counting the number of lesions for session 1 or 2; or a sensitivity of 90.7% or 93.0% for session 1 or 2 and a specificity of 98.6% for both sessions when counting the number of patients with lesions. Reader 1 counted one false-positive lesion in both reading sessions, and reader 2 counted four false-positive lesions when reading only post-contrast T1-w images, in contrast to three false-positive lesions when reading T1-w images pre- and post-contrast. No significant differences were found between both reading sessions (reader 1, *p* = 1.000; reader 2, *p* = 0.317). The slightly higher sensitivity for reader 1 might be attributed to the longer experience in image reading. In consensus, no hyperintense signal on post-contrast T1-w images was found without a corresponding hyperintense signal on T2-w images, which means no false-positive ce lesions were observed. 37/43 (86%) of these patients were diagnosed with (suspected) MS, 5/43 (12%) patients with CIS, and 1/43 (2%) with NMOSD.

**Table 3:** Contingency tables with numbers of contrast-enhancing lesions given separately per reader and session

Number of lesions (T2 lesions: *n* = 745; contrast-enhancing lesions: *n* = 56)				Number of patients with lesions (patients with T2 lesions: *n* = 183; patients with contrast-enhancing lesions: *n* = 43)			
Reader 1	Session 1	Rd 1 CE−	Rd 1 CE+	Reader 1	Session 1	Rd 1 CE−	Rd 1 CE+
	Consens Gd−	688	1		Consens Gd−	139	1
	Consens Gd+	1	55		Consens Gd+	1	42
	Session 2	Rd 1 CE−	Rd 1 CE+		Session 2	Rd 1 CE−	Rd 1 CE+
	Consens Gd−	688	1		Consens Gd−	139	1
	Consens Gd+	1	55		Consens Gd+	1	42
Reader 2	Session 1	Rd 2 CE−	Rd 2 CE+	Reader 2	Session 1	Rd 2 CE−	Rd 2 CE+
	Consens Gd−	686	3		Consens Gd−	138	2
	Consens Gd+	4	52		Consens Gd+	4	39
	Session 2	Rd 2 CE−	Rd 2 CE+		Session 2	Rd 2 CE−	Rd 2 CE+
	Consens Gd−	686	3		Consens Gd−	138	2
	Consens Gd+	3	53		Consens Gd+	3	40

*p* = 1 (reader 1), *p* = 0.317 (reader 2). *ce/CE* contrast-enhancing/contrast enhancement, *Rd* reader, *Session 1* T1-w post-contrast only, *Session 2* T1-w pre- and post-contrast, *Consens* consensus reading between both readers as gold standard

No significant differences in diagnostic confidence were found for both readers between both reading sessions when excluding or including pre-contrast T1-w images: reader 1: 4.54 ± 0.76, range 2–5 (session 1) versus 4.66 ± 0.59, range 3–5 (session 2), *p* = 0.058; reader 2: 4.46 ± 0.84, range 2–5 (session 1) versus 4.44 ± 0.86, range 2–5 (session 2), *p* = 0.317. Intra-rater concordance was moderate to substantial (*κ* = 0.699) for reader 1 and almost perfect (*κ* = 0.962) for reader 2.

There were no significant differences regarding the image assessment between both readers except for overall image quality: artifacts: 1.24 ± 0.70, range 1–4 (reader 1) versus 1.27 ± 0.68, range 1–4 (reader 2), *p* = 0.343; overall image quality: 1.46 ± 0.67, range 1–5 (reader 1) versus 1.63 ± 0.70, range 1–4 (reader 2), *p* < 0.001; visual CE: 2.04 ± 0.83, range 1–3 (reader 1) versus 2.06 ± 0.82, range 1–3 (reader 2), *p* = 0.564. The inter-rater concordance between both readers was moderate regarding artifacts (*κ* = 0.418) and overall image quality (*κ* = 0.504) or regarding visual CE (*κ* = 0.504). Eleven patients were rated by a score of 4 (“poor”) or 5 (“inappropriate”) in the category overall image quality due to considerable motion artifacts in post-contrast T1-w images.

## Discussion

This study revealed that diagnostic accuracy and confidence for the evaluation of ce lesions in the spinal cord of patients suffering from MS or related syndromes were comparable when assessing only post-contrast T1-w images compared to the assessment of both pre- and post-contrast T1-w images. This is in accordance with current recommendations for spinal MRI protocols, which suggest to not necessarily include a pre-contrast T1-w sequence by default [5, 6]. These recommendations, however, have not been proven by a systematic study to date.

When contrast medium is needed, usually a pre-contrast T1-w sequence is acquired in addition to post-contrast T1-w sequences for proper assessment of a real CE. Hence, the question arises whether the exclusion of pre-contrast T1-w images in spinal MRI for patients with MS is justifiable and would come without drawbacks for diagnostic performance and confidence. There are some pitfalls imitating a CE that a radiologist should be aware of. A shortening of the T1 signal can be caused by other pathologies such as hemorrhage or fat. In the rather homogenous structure of the spinal cord, the presence of fat or blood is, however, very rarely seen. In our study, no false-positive lesions were found in consensus reading in post-contrast T1-w images when assessing T2-w images in parallel. In detail, both readers counted few false-positive lesions in each reading session, however without significant differences between both reading sessions. These false-positive lesions could be attributed in retrospect to artifacts at the outer contour of the cervical spinal cord, possibly due to swallowing. Furthermore, a black hole might lead to only a subtle CE in post-contrast T1-w images and might influence diagnostic confidence. In these cases, a comparison with a non-contrast scan is helpful. However, the incidence of hypointense spinal cord lesions on T1-w images prior to contrast administration is a rather seldom finding in MS patients and is more common in patients with a more progressive disease course or a longer disease duration [5]. They are also more often attributed to Devic’s neuromyelitis optica [2, 9] or even to the presence of a tumor [8, 10]. T1-w hypointense lesions can occur in the chronic phase of NMOSD patients, possibly representing cystic changes [8]. Previously, black holes in the spinal cord were seen in only 1/104 (1%) of patients with MS [2] or in 9% of the lesions [11]. In our study, we found hypointensities on T1-w images in 31/745 (4%) lesions or in 23/244 (9%) patients with MS or CIS, and in 2/8 (25%) patients with NMOSD. Even with these slightly higher numbers of prevalences of black holes, both readers managed to detect ce lesions with high diagnostic confidence when excluding pre-contrast T1-w images.

As discussed by Gass et al [11], the reason for the rare finding of black holes in the spinal cord might be caused by a different biological pattern of the lesions or the small size of the lesions and the limited sensitivity of spinal MRI, which is due to restricted image resolution and partial volume effects. A few studies have been published in the last years, showing a good correlation between hypointensities on T1-w images of the spinal cord and clinical presentation of disability of the patients [12, 13]. Other studies analyzed the techniques’ sensitivities and recommended an optimized T1-w magnetization-prepared rapid gradient echo (MPRAGE) sequence [14] or a phase-sensitive T1-w sequence [15, 16]. However, T1-w hypointense lesions in the spinal cord have not been established as criteria in the MRI guidelines for MS [5], which is probably due to the technical difficulties of spinal cord imaging. These considerations and the results of our study might be helpful for the decision whether to omit pre-contrast T1-w images in spinal MRI for follow-up investigations of patients with MS.

Furthermore, our results regarding hyperintense spinal cord abnormalities on T2-w imaging are in concordance with a study analyzing the spinal cord in recently diagnosed MS [2]. We found T2-w hyperintense lesions in about 69% of all patients, compared to 83% among patients with recently diagnosed MS in this previous study [2]. Specifically, 53% of the lesions were located in the cervical spinal cord, as compared to 56.4% in the literature [2]. Furthermore, CE was described in up to 17.2% [2], compared to 16.2% of the patients of our study (7.5% of the lesions).

As described above, eleven patients showed considerable motion artifacts in post-contrast images. Pre-contrast images of all patients, however, showed no relevant motion artifacts. In these cases, image quality and diagnostic value would probably have been more appropriate when excluding pre-contrast images to shorten scan duration and increase patient compliance. Future studies with a prospective approach, ideally including a multi-centric design, may confirm our findings.

We acknowledge some limitations of our retrospective study. The delay between the administration of Gd and the acquisition of post-contrast T1-w sequences was not equal for all patients, resulting in a range of 4.0–8.5 min with a median of 6.2 min. However, Uysal et al [17] concluded that there is no significant difference in lesion numbers with T1-w images acquired 5 or 10 min after the administration of Gd. We focused on lesions within the spinal cord; yet, pre-contrast images can be helpful to diagnose incidental findings especially in the vertebral bodies. Finally, a very small number of MS lesions might show T1-hyperintensities on pre-contrast T1-w images, which is probably due to the presence of lipid- and iron-laden microglia/macrophages, abnormal accumulation of proteins, or paramagnetic free radicals [18, 19]. T1-hyperintense lesions on pre-contrast T1-w images are supposed to be found particularly in the chronic stages of MS, and CE of the lesions can be seen after treatment with long-lasting blood-brain barrier disruption and does not always indicate active lesions. This has to be considered to prevent overtreatment. In these rare cases, pre-contrast T1-w images can help to discriminate this cause of T1-hyperintensity from true CE.

In conclusion, acquisition of pre-contrast T1-w images does not significantly increase diagnostic confidence or detection rate of ce lesions in the spinal cord in patients with (suspected) MS. Pre-contrast T1-w sequences might therefore be excluded from spinal MRI for follow-up examinations in patients with MS, thus probably providing more time for other sequences to improve diagnostic yield or to enhance the patients’ compliance by reducing scan time. This does however not exclude the necessity to perform pre-contrast T1-w scans in special clinical situations in which certainty on CE is required.

## Abbreviations

CE: Contrast enhancement
ce: Contrast-enhancing
CIS: Clinically isolated syndrome
CNS: Central nervous system
DIR: Double inversion recovery
DIS: Dissemination in space
DIT: Dissemination in time
Gd: Gadolinium
*κ*: Cohen’s kappa
MPRAGE: Magnetization-prepared rapid gradient echo
MRI: Magnetic resonance imaging
MS: Multiple sclerosis
NMOSD: Neuromyelitis optica spectrum disorders
PACS: Picture archiving and communication system
RIS: Radiologically isolated syndrome
STIR: Short tau inversion recovery
TSE: Turbo spin echo

## Declarations

### Guarantor

The scientific guarantor of this publication is Jan S. Kirschke, MD.

### Statistics and biometry

One of the authors has significant statistical expertise.

### Methodology

• retrospective

• diagnostic or prognostic study

• performed at one institution
